# Smoking and COVID-19: Did we overlook representativeness?

**DOI:** 10.18332/tid/129584

**Published:** 2020-11-02

**Authors:** Thomas Wenzl

**Affiliations:** 1 European Commission, Joint Research Centre (JRC), Geel, Belgium

**Keywords:** smoking, COVID-19, nicotine

The risk factors for contracting symptomatic COVID-19 are not fully understood yet. Age and certain underlying health conditions are considered to be detrimental for disease outcomes. The World Health Organisation associated smoking with an adverse progression of the disease and called on smokers to quitting smoking. However, a review of 174 cohort studies revealed an unexpected low number of current smokers among subjects tested for SARS-CoV-2 infections^1^. The prevalence of current smokers suffering from symptomatic COVID-19 was frequently significantly lower than in the general population. Current smokers were at reduced risk of being tested positive compared to former smokers and never smokers, which might have been caused by different testing frequencies, but were at higher risk for severe symptomatic COVID-19^1^. This low prevalence of current smokers among COVID-19 patients led to the hypothesis that smoking/ nicotine uptake might have a preventive effect^2,3^. Research was initiated on the interrelation of nicotine, the renin angiotensin system (RAS) and SARS-CoV-2 infections. Amongst others, the downregulation of the expression of angiotensinconverting enzyme 2 (ACE-2) as well as an inhibitory effect on the production of pro-inflammatory cytokines were identified as potential effects of exposure to nicotine. This is difficult to understand seeing that other studies found an increased expression of ACE-2 in smokers, the entrance gate of the coronavirus into human cells^4,5^. In the evaluation of the cohort studies, little attention was given to the possibility that the use of the proportion of smokers in the general population as a reference for deriving prevalence ratios to study the association of smoking with COVID-19 disease outcomes may be inappropriate. Prevalence data for smoking and comorbidities (hypertension, diabetes mellitus, and chronic obstructive pulmonary disease) reported in 25 studies, which partially identified a potentially beneficial effect of smoking/nicotine intake, were re-analysed to investigate the relationship between symptomatic COVID-19 and national smoking prevalence taking account of known risk factors associated with the disease (Supplementary file)^6^. The limited agreement of the prevalence of those risk factors in the general population with the cohort data demonstrates indirectly that these patients most likely do not reflect the health status of the general population. In the absence of specifically designed studies, any hypothesis on the effect of smoking/nicotine uptake on symptomatic COVID-19 remains speculative. The number of potentially confounding variables would require a multivariate statistical approach and large cohort sizes for providing clarity on the significance of potential effects. However, the structure of the published aggregated data permits only univariate approaches. As such, the hypothesis of a potentially protective effect of smoking/nicotine uptake on symptomatic COVID-19 cannot be verified.

## CONFLICTS OF INTEREST

The author has completed and submitted the ICMJE Form for Disclosure of Potential Conflicts of Interest and none was reported.

## FUNDING

There was no source of funding for this research.

## PROVENANCE AND PEER REVIEW

Commissioned; internally peer reviewed.
