# Supplementary material for: Smoking and COVID-19: Did we overlook representativeness?
Source: Tob Induc Dis. 2020 Nov 2;18:89. doi: 10.18332/tid/129584 (PMC7605409; doi:10.18332/tid/129584)
Supplement: Supplementary file 1 [file TID-18-89-s1.pdf]

European Commission

JRC TECHNICAL REPORT

Smoking and COVID-19

A review of studies suggesting a protective effect of smoking against COVID-19

Wenzl, T

2020

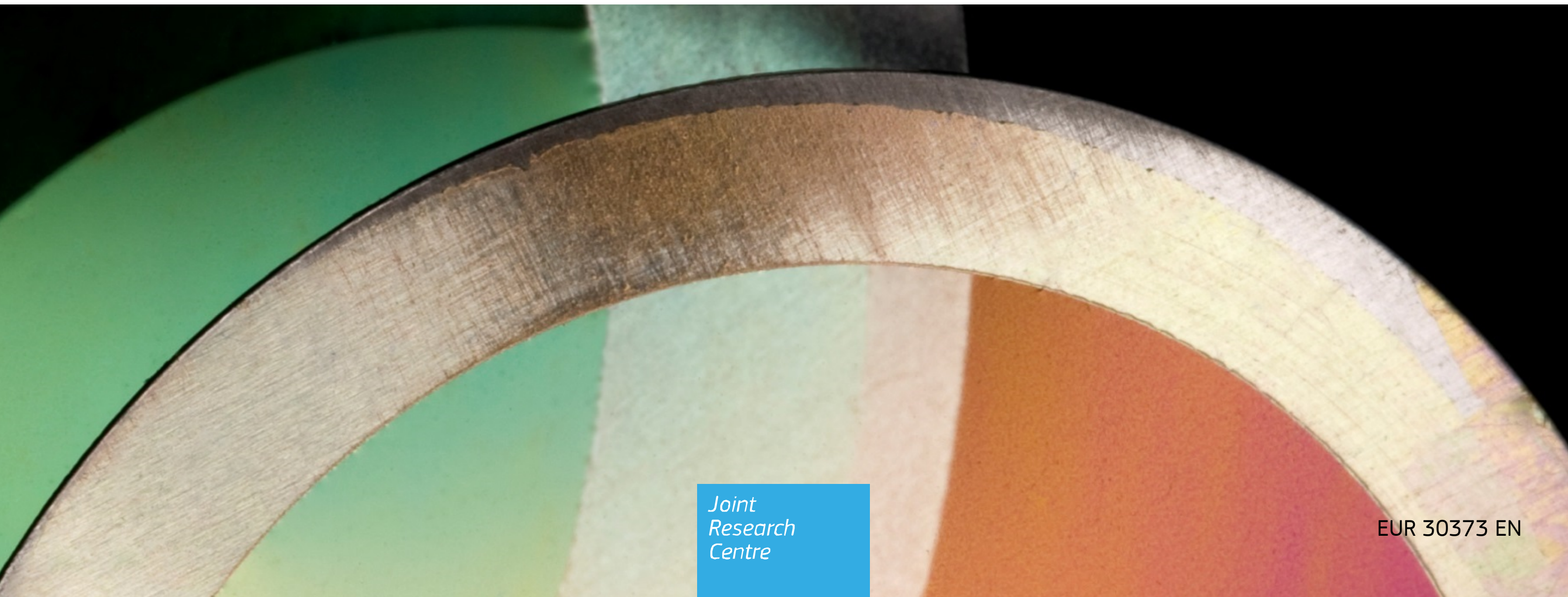

Joint Research Centre

EUR 30373 EN

This publication is a Technical report by the Joint Research Centre (JRC), the European Commission's science and knowledge service. It aims to provide evidence-based scientific support to the European policymaking process. The scientific output expressed does not imply a policy position of the European Commission. Neither the European Commission nor any person acting on behalf of the Commission is responsible for the use that might be made of this publication. For information on the methodology and quality underlying the data used in this publication for which the source is neither Eurostat nor other Commission services, users should contact the referenced source. The designations employed and the presentation of material on the maps do not imply the expression of any opinion whatsoever on the part of the European Union concerning the legal status of any country, territory, city or area or of its authorities, or concerning the delimitation of its frontiers or boundaries.

Contact information
Name: Thomas Wenzl
Address: Joint Research Centre Geel
Email: thomas.wenzl@ec.europa.eu

EU Science Hub
https://ec.europa.eu/jrc

JRC121837

EUR 30373 EN

PDF ISBN 978-92-76-22062-6 ISSN 1831-9424 doi:10.2760/564217

Luxembourg: Publications Office of the European Union, 2020

© European Union, 2020

The reuse policy of the European Commission is implemented by the Commission Decision 2011/833/EU of 12 December 2011 on the reuse of Commission documents (OJ L 330, 14.12.2011, p. 39). Except otherwise noted, the reuse of this document is authorised under the Creative Commons Attribution 4.0 International (CC BY 4.0) licence (https://creativecommons.org/licenses/by/4.0/). This means that reuse is allowed provided appropriate credit is given and any changes are indicated. For any use or reproduction of photos or other material that is not owned by the EU, permission must be sought directly from the copyright holders.

All content © European Union 2020

How to cite this report: Wenzl, T., *Smoking and COVID-19 – A review of studies which motivated unexpected health claims*, EUR 30373 EN, Publications Office of the European Union, Luxembourg, 2020, ISBN 978-92-76-22062-6, doi:10.2760/564217, JRC121837.

Contents

Summary 2
Introduction 3
Nicotine, the Renin-Angiotensin System and COVID-19 4
Smoking and COVID-19 5
Exploratory data analysis 10
Findings 11
References 13

Summary

The risk factors for contracting symptomatic COVID-19 are not yet fully understood, age and certain underlying health conditions are considered to be detrimental in this respect. Case studies revealed an astonishingly low number of current smokers among patients suffering from symptomatic COVID-19 compared to the general population, leading to the conclusion that smoking/nicotine uptake might have a preventive effect. This is difficult to understand seeing that studies found an increased expression of the angiotensin-converting enzyme (ACE-2) in smokers, the entrance gate of the coronavirus into human cells. Consequently, the use of the proportion of smokers in the general population as a reference for deriving prevalence ratios to study the association of smoking with COVID-19 disease outcomes may be inappropriate. Prevalence data for smoking and comorbidities (hypertension, diabetes mellitus, and chronic obstructive pulmonary disease) reported in 25 studies, which partially identified a potentially beneficial effect of smoking/nicotine intake, were re-analysed to investigate the relationship between COVID-19 mortality and national smoking prevalence taking account of known risk factors associated with mortality. The limited agreement of the prevalence of those risk factors in the general population with the cohort data demonstrates indirectly that these patients most likely do not reflect the health status of the general population. In the absence of specifically designed studies, any hypothesis on the effect of nicotine on symptomatic COVID-19 remains speculative. The number of potentially confounding variables would require a multivariate statistical approach and large cohort sizes for providing clarity on the significance of potential effects. However, the structure of the published aggregated data permits only univariate approaches. As such, the hypothesis of a potentially protective effect of nicotine on symptomatic COVID-19 cannot be verified.

Introduction

The conclusions from a cross sectional study conducted by French scientist that smoking might protect against symptomatic infections with the severe acute respiratory syndrome coronavirus 2 (SARS-CoV-2) has caused both a lot of attention in public media and shockwaves among tobacco control organisations (1). The response of the public, exposed to uncertainty and fear about SARS-CoV-2 infections, was so massive, that the French Ministry of Health imposed on 23 April 2020 temporary restrictions on the sale of nicotine supplying therapeutics, which are usually used in smoking cessation therapy (2, 3). The respective ministerial decree reasoned the measure with prevention of self-medication and overdosing of nicotine, and ascertaining an uninterrupted supply of the products for therapeutic purposes. There is also anecdotal information about an increase in cigarette prices in Iran, due to an increased demand of tobacco products after the publication of the French study. France, however, declared at the virtual meeting of the G7 health ministers to further study the potentially positive effect of nicotine in fighting the coronavirus disease 2019 (COVID-19).

It is without surprise that the tobacco control community issued warnings and advised strongly against (commencing) smoking for preventing COVID-19.

Questions, which have to be addressed in this context, are whether smoking, respectively nicotine uptake, have any effect on COVID-19, and if so, what is the magnitude of the effect. Press reports relating the low number of smokers with symptomatic COVID-19 to beneficial effects of smoking do not properly reflect the current scientific debate, which centres on physiological effects of nicotine uptake and not on smoking.

It has to be noted that most publications on the possible link between smoking (nicotine consumption) and COVID-19 outcomes provide plausible hypotheses, but lack experimental evidence. Much of the relevant information is still under peer review and published on pre-print portals only.

Nicotine, the Renin-Angiotensin System and COVID-19

There is large agreement in the scientific community that SARS-CoV-2 enters host cells via the angiotensin-converting enzyme 2 (ACE2), a transmembrane protein with both extracellular and intracellular components (4-7). ACE2 is part of the renin-angiotensin system (RAS), which exerts different functions in the human body, among them it is involved in the regulation of blood pressure (8). Downregulation of ACE2 in virus-infected cells triggers a response of the immune system, including the production of pro-inflammatory cytokines, which can lead to a so-called 'cytokine storm' resulting in multi-organ failure and, ultimately, leading to death (6).

Any intervention in the homeostasis of the RAS system may have consequences regarding the susceptibility to SARS-CoV-2 infections and outcomes of COVID-19. Pharmaceutical interventions and environmental as well as individual behavioural factors, such as nicotine consumption via smoking, could intervene in RAS homeostasis (9, 10).

Nicotine binds in the human body to the nicotinic acetylcholine receptor, which is expressed in many body tissues. Several biochemical mechanisms are used to explain a hypothetical effect of nicotine on COVID-19 outcomes and the low prevalence of smokers among hospitalised COVID-19 patients:

- A nicotine dependent downregulation of the expression of the ACE2 receptor in several body tissues could limit the number of entry gates for SARS-CoV-2.
- The inhibitory effect of nicotine on the production of pro-inflammatory cytokines, which led to adverse outcomes in COVID-19 patients if released in overwhelming amounts ("cytokine storm").
- The immune system of current smokers might be more tolerant and less prone to the over-production of immune cells and cytokines compared to immunocompetent non-smokers, reducing thereby the likelihood of the development of acute respiratory distress syndrome (ARDS).

Contrary to the described effects, several authors found higher ACE2 gene expression levels in small airway epithelial cells of smokers and COPD patients compared to former smokers and never smokers (11, 12).

Smoking and COVID-19

The basis for triggering a debate regarding a possible beneficial effect of smoking is formed by several retrospective studies on clinical characteristics and comorbidities of hospitalized COVID-19 patients (1, 13-37).

Earlier meta-analyses aimed to elucidate the effect of smoking on the prevalence and severity of COVID-19 (35, 38-43). They analysed studies with large differences in cohort sizes and inconsistent endpoints (Table 1). Only Vardavas and Nikitara (Relative Risk[1] (RR)=2.4; 95% CI 1.43-4.04) and Farsalinos et al. (Odds Ratio[2]=1.53; 95% CI 1.06-2.20) demonstrated that the severity of the illness and the fatality rate of active smoker hospitalised for COVID-19 is worse compared to non-smokers (38, 43). The majority of meta-analyses did not identify statistically significant effects, or only a questionable effect of smoking on the severity of COVID-19 (39-41). The latter was observed in a meta-analysis of case studies by Zhao et al., who reported for active smokers a doubling of the risk to develop sever COVID-19 compared to non-smokers (weighted odd ratio of about two), which vanished after breaking down and compiling studies according to differences in endpoints (40).

Contrary to meta-analyses that resulted in either no observable effects of smoking or a negative influence on the progression of COVID-19, several meta-analyses pointed out that the number of smokers among hospitalised COVID-19 patients was low in comparison to the smoking habits of the underlying general population. The very same was reported by Simons et al. (44) in their most recent version of the living rapid evidence review on the association of smoking status with SARS-CoV-2 infection, hospitalisation and mortality from COVID-19. The sixth version of this living rapid evidence review compiles information from 174 observational studies stratified by smoking status.

[1] RR compares the risk of a health event (disease or death) among one group with the risk among another group. A risk ratio of 1.0 indicates identical risk among the two groups.

[2] OR represents the odds that an outcome will occur given a particular exposure, compared to the odds of the outcome occurring in the absence of that exposure.

Table 1: Overview on meta-analyses

	Studies	Subjects	Former Smoker	Endpoint	Indicator	Pooled Effect*
**Lippi et al. (39)**	5	1399	No information	Severe/non-severe COVID-19	OR	OR=1.69 (0.41-6.92)
**Vardavas & Nikitara (38)**	5	1549		1) Severe/non-severe COVID-19,<br>2) ICU admission, mechanical ventilation, death	1) RR<br>2) RR	1) RR=1.4 (0.98-2.00)<br>2) RR=2.4 (1.43-4.04)
**Farsalinos Barbouni Niaura (42)**	13	5960	1) Left out<br>2) Pooled with smoker	Smoking prevalence	P	1) P=6.5% (4.9%-8.2%)<br>2) P=7.3% (5.7%-8.9%)
**Farsalinos et al. (43)**	18	6515	Included in non-current smoking	1) Current smoking<br>2) Adverse outcome	1) POR<br>2) OR	1) POR=0.20 (0.16-0.25)<br>2) OR=1.53 (1.06-2.20)
**Zhao et al. (40)**	7	1726	Included in non-smoker	Severity of COVID-19	OR	OR=1.97 (0.95-0.27)
	1) 4	1) 1216		1)Subgroup ISU, Ventilation or death	1) OR	1) OR=1.43 (0.49-4.22)
	2) 3	2) 329		2)Severe COVID-19 or disease progression	2) OR	2) OR=2.86 (0.73-11.24)
**Gonzales Rubio et al. (41)**	18	7671	Included in non-smoker	Smoking prevalence	OR	OR=0.18 (0.14-0.22)

OR: Odds ratio, POR: Prevalence odds ratio, P: Prevalence, RR: Relative risk

* 95% confidence intervals given within brackets

The outcomes of the published meta-analyses have to be interpreted with caution, as different assumptions were made about the prevalence of non-smokers in the different cohorts, which was caused by the lack of explicit data on former smokers and non-smokers. Consequently, subjects not identified as smoker were considered as non-smoker. If data on former smokers were available, they were grouped partially with current smokers (42) and partially with non-smokers (40, 43). The meta-analyses used also different indicators for the evaluation and interpretation of the studies. A common limitation to all presented meta-analyses is the fact that most of the data stem from retrospective case studies, which did not consider confounding variables. Simons et al. identified a number of issues, which could introduce bias respectively complicate at least the interpretation of the observational studies (44). The selection of the subjects included in the published studies occurred according to criteria such as hospitalisation, development of severe pneumonia, or other endpoints. Additionally, the double accounting of patient data in different studies cannot be excluded, e.g. the two publications of Guan et al. comprise likely overlapping study cohorts (13, 14). The same is expected for data published by the CDC, and by Goyal et al., which concern geographically overlapping areas (30, 37).

The characteristics of the analysed cohorts as well as the reported smoking status is summarized in Table 2. All cohorts had lower numbers of smokers in comparison to the number of smokers in the related population. The calculated prevalence ratios (PR) were with two exceptions significantly below unity. This data is irritating, as it is reasonable to expect that outcomes from COVID-19 infection are worse for smokers, as is the case in other acute respiratory infections.

Patients in most of the studied cohorts were of high average age, triggering the question whether smoking prevalence data of the general population forms a valid basis for making comparisons. Although none of the studies explicitly claimed that the investigated cohorts are representative for the general population. Providing further evidence that the studied cohorts reflect health related conditions of the general population could be useful for substantiating the potential effect of nicotine uptake on the progression of COVID-19 in hospitalised patients.

This question was approached by investigating whether data on comorbidities reported in the cohort studies correlate with the related data of the underlying populations. Data on the non-communicable diseases hypertension, diabetes mellitus, and chronic obstructive pulmonary disease (COPD) were extracted from the cohort studies and combined with published prevalence data for these illnesses. This allowed estimating the theoretical number of comorbidity cases in the studied cohorts if prevalence rates of the general population were assumed. The investigation was centred on 25 studies forming the basis for early meta-analyses, which partially identified a potentially beneficial effect of smoking/nicotine intake.

Table 2: Observed and expected smoker, prevalence ratio (PR), and probability that observed population is representative for general population

Author	Country	Obs.	Male	Female	Smoker obs.	Smoker ratio obs.	95% CI, LL	95% CI, UL	Smoker exp.	Smoker ratio exp.	PR	P-Value
		#			#				#			
**Zhang et al. (16)**	CN	133	0.507	0.493	2	0.015	0.002	0.053	37	0.278	0.05	0.000
**Huang et al. (18)**	CN	41	0.732	0.268	3	0.073	0.015	0.199	16	0.390	0.19	0.000
**Guan, Ni et al. (14)**	CN	1099	0.582	0.418	137	0.125	0.106	0.146	346	0.315	0.40	0.000
**Yang et al. (15)**	CN	52	0.673	0.327	2	0.038	0.005	0.131	19	0.365	0.11	0.000
**Guan, Liang et al (13)**	CN	1590	0.569	0.424	111	0.070	0.058	0.084	489	0.308	0.23	0.000
**Chen et al. (19)**	CN	274	0.624	0.376	7	0.026	0.011	0.053	92	0.336	0.08	0.000
**Liu et al. (17)**	CN	78	0.500	0.500	5	0.064	0.021	0.143	21	0.269	0.24	0.000
**Zhou et al. (20)**	CN	191	0.623	0.377	11	0.058	0.029	0.101	64	0.335	0.17	0.000
**Zhang, Cai et al. (23)**	CN	645	0.509	0.491	41	0.064	0.046	0.086	179	0.278	0.23	0.000
**Wang et al. (27)**	CN	125	0.568	0.432	16	0.128	0.075	0.200	38	0.304	0.42	0.000
**Wan et al. (22)**	CN	135	0.533	0.467	9	0.067	0.031	0.123	39	0.289	0.23	0.000
**Shi et al. (24)**	CN	474	0.532	0.468	40	0.084	0.061	0.113	141	0.297	0.28	0.000
**Feng et al. (28)**	CN	454	0.569	0.431	44	0.097	0.071	0.128	147	0.324	0.30	0.000
**Ji et al. (29)**	CN	208	0.563	0.438	19	0.091	0.056	0.139	63	0.303	0.30	0.000
**Li et al. (26)**	CN	544	0.509	0.491	41	0.075	0.054	0.100	153	0.281	0.27	0.000
**Mo et al. (21)**	CN	155	0.555	0.445	6	0.039	0.015	0.083	47	0.303	0.13	0.000
**Kim et al. (25)**	KR	28	0.536	0.464	5	0.179	0.061	0.369	6	0.214	0.83	0.855
**Goyal et al. (30)**	US	393	0.606	0.394	20	0.051	0.031	0.078	83	0.211	0.24	0.000
**CDC (37)**	US	7162	-	-	96	0.013	0.011	0.016	1411	0.197	0.07	0.000
**Gold et al. (36)**	US	305	0.495	0.505	16	0.052	0.030	0.083	61	0.200	0.26	0.000
**Miyara et al. (1)***	FR	341	0.601	0.399	21	0.062	0.039	0.093	89	0.259	0.24	0.000
**Miyara et al. (1)****	FR	132	0.460	0.547	13	0.098	0.054	0.160	35	0.252	0.39	0.000
**Miyara et al. (1)*****	FR	473	0.560	0.442	34	0.072	0.050	0.099	125	0.259	0.28	0.000
**Han et al. (31)**	CN	17	0.353	0.647	3	0.176	0.038	0.434	3	0.176	1.00	1.000
**Jin et al. (32)**	CN	651	0.492	0.508	41	0.063	0.046	0.084	176	0.270	0.23	0.000

**Lian et al. (33).**	CN	788	0.516	0.484	54	0.069	0.052	0.089	222	0.282	0.24	0.000
**Yao et al. (34)**	CN	108	0.398	0.602	4	0.037	0.010	0.092	24	0.222	0.17	0.000
**Zhang, Ouyang et al. (35)**	CN	120	0.358	0.642	6	0.050	0.019	0.106	24	0.200	0.25	0.000

[1] hospitalized patients; [2] non-hospitalized patients; [3] all patients;

PR: prevalence ratio; obs.: observed; exp.: expected; 95% CI: 95 % confidence interval; LL: lower limit; UL: upper limit

Exploratory data analysis

Information on the smoking status and comorbidities of the investigated cohorts were compiled from the respective publications. Prevalence ratios for smoking were calculated for the studied cohorts taking into account the actual number of subjects for which information on smoking status was specified. The expected prevalence ratios for active smokers were determined for the given cohort sizes respecting gender and country dependent differences. Actual data on the smoking status and prevalence of hypertension, diabetes mellitus and COPD in the respective countries was retrieved from public sources (Table 3). Hypertension rates for Chinese cohorts were adjusted for gender and geographical region applying rates specified in the electronic supplement of Wang et al. (45). The expected hypertension prevalence of subjects from 30 respectively 31 provinces were estimated with hypertension rates for the general Chinese population, whereas averages of province dependent rates were used in case data comprised subjects from more than one province (45). Country-specific diabetes prevalence data were retrieved for China, France and Korea from the International Diabetes Federation (IDF), which however did not allow discriminating according to gender, and from Virani et al. for the USA (46, 47). Chronic obstructive pulmonary disease (COPD) occurs mainly at elderly people. For this reason prevalence data are often provided only for certain age groups, such as Chinese and Korean adults above 40 years of age (48, 49). Applying them to the general population will likely overestimate the prevalence of COPD in that country. Additionally, available prevalence data are associated with significant uncertainties and might be subject to geographical variations (50). It should also be noted that some COPD prevalence data is already more than a decade old and might not anymore reflect the current situation (51). Acknowledging these limitations, they might nonetheless be applicable to the studied cohorts as the average age of the cohorts was mostly high as well. Most recent US countrywide prevalence data was preferred to older federal state specific prevalence data (50, 52).

Table 3: Smoking prevalence and prevalence of comorbidities in the countries of the studied cohorts

	**Smoking**		**Hypertension**		**Diabetes mellitus**		**COPD**	
**Data sources**	(53-56)		(45, 47, 57, 58)		(46, 47)		(48-51)	
**Gender**	M	F	M	F	M	F	M	F
**Country/Region**	%	%	%	%	%	%	%	%
FR	28.2	22.9	36.5	25.2	7.6		7.5	
KR	35.8	6.5	35.0	22.9	9.2		21.6	5.8
US	25.8	14.1	49.0	42.8	10.9	8.9	6.4	
CN	52.1	2.7			10.9		19	8.1
CN- Hubei			19.7	16.5				
CN- Chongqing			20.4	20.7				
CN- Zhejiang			25.4	21.0				
CN- Anhui, Hubei, Shanghai			23.8	21.3				
CN- 30/31 provinces			24.5	21.9				

M: male; F: female

FR: France; KR: South Korea; US: United States of America; CN: People's Republic of China

Expected occurrence figures and occurrence rates were calculated for the studied cohorts taking into account the respective cohort size and prevalence data given in Table 3. Occurrence figures were rounded to the next integer. Occurrence rates calculated for the studied cohorts were complemented by their 95% confidence intervals. Statgraphics Centurion 18 (Statgraphics Technologies Inc) was applied for that purpose, as well as for deriving respective probability values.

Findings

Exploratory data analysis confirmed the mismatch of the observed number of smokers with the number of smokers expected for the cohort size in the general population. Prevalence ratios for smoking were with the exception of two studies significantly below one and p-values usually close to zero. These observations formed the basis for the hypothesis that nicotine uptake might have a protective effect against symptomatic COVID-19. However, the figures demonstrate only a difference between the number of observed smokers and the number of smokers expected for the particular cohort. Nonetheless, the plausibility of the hypothesis of a protective effect of nicotine against symptomatic COVID-19 would be supported if it could be demonstrated that prevalence in the general population of other medical conditions is reflected in the published cohort data.

The investigation of hypertension, diabetes, and COPD revealed for many cohorts a statistically significant discrepancy between the observed prevalence and the prevalence in the general population. As for smoking, the observed prevalence was frequently lower than the one expected in the general population. A compilation of prevalence ratios is provided in Table 4. Figures in red indicate at the 95 % confidence level a significantly lower prevalence observed in the studied cohorts compared to the prevalence expected in the general population of the respective country, figures in green indicate the opposite, and figures in black were not statistically significant (95% confidence level) different from the general population.

The power of the applied binomial test is strongly influenced by the size of the cohort. The smaller it is the lower is the power of the test, which explains the lack of statistical significance of some prevalence ratios largely different from one in either direction (Table 4). The situation changes with large cohort sizes, for which reason it is even more astonishing that the patients in the largest cohorts did not match the prevalence for any of the four features in the general population. This, however, must be interpreted with caution, as some cohort data might not be independent from each other, e.g. papers published by Lian et al., Jin et al., and Zangh, Cai et al. comprise patient data from the same hospital in Hangzhou (province Zhejiang, China) (23, 32, 33); this is also likely the case for papers published by Guan, Ni et al. and Guan, Liang et al. (13, 14). Even if the potential repetition of patient data is taken into account, the health status of these cohorts appear to be considerably better than in the underlying population, despite suffering from symptomatic COVID-19. The same is observed for patients from the United States, for which information on two comorbidities was not specific enough to be included in this evaluation. As mentioned by the authors, the quality of data might be compromised by the rapid evolution of the pandemic, urgency of medical interventions, and lack of resources, which might explain the lack of complete data on underlying health conditions in more than 94 % of the more than 122000 studied case reports. Therefore, representativeness of the data reported in the different studies for the general population cannot be presumed and caution has to be exercised in the interpretation of the outcomes of some meta-analyses due to this limitation and the mentioned potentially double accounting of patient data.

The limited agreement of the reported cohort data with the general population in terms of prevalence of underlying health conditions other than smoking demonstrates indirectly that these patients most likely do not reflect the situation of the general population. In the absence of specifically designed studies, any hypothesis on the effect of nicotine on symptomatic COVID-19 remains speculative. The number of potentially confounding variables would require a multivariate statistical approach and large cohort sizes for providing clarity on the significance of potential effects. However, the structure of the published aggregated data permits only univariate approaches. As such, the hypothesis of a potentially protective effect of nicotine on symptomatic COVID-19 cannot be verified. Consequently, specially designed studies are warranted for elucidating the effect of smoking/nicotine uptake on the development of symptomatic COVID-19.

Table 4: Compilation of prevalence ratios (PR) of the observed prevalence with the prevalence in the respective general population

Author	Country	Obs.	Age*	PR			
		#	y	Smoking	HT	Diabetes	COPD
**Zhang et al. (16)**	CN	133	57	0.05	1.66	1.11	0.11
**Huang et al. (18)**	CN	41	49	0.19	0.78	1.79	0.15
**Guan, Ni et al. (14)**	CN	1099	47	0.40	0.64	0.68	0.08
**Yang et al. (15)**	CN	52	59.7	0.11		1.59	0.50
**Guan, Liang et al (13)**	CN	1590	48.9	0.23	0.73	0.75	0.11
**Chen et al. (19)**	CN	274	62	0.08	1.83	1.57	
**Liu et al. (17)**	CN	78	38	0.24	0.57	0.59	0.19
**Zhou et al. (20)**	CN	191	56	0.17	1.64	1.73	0.21
**Zhang, Cai et al. (23)**	CN	645	45.2	0.23	0.67	0.68	0.01
**Wang et al. (27)**	CN	125	38.8	0.42			
**Wan et al. (22)**	CN	135	47	0.23	0.47	0.82	0.21
**Shi et al. (24)**	CN	474	46	0.28	0.87	0.55	
**Feng et al. (28)**	CN	454	53	0.30	1.04	0.94	
**Ji et al. (29)**	CN	208	44	0.30			
**Li et al. (26)**	CN	544	60	0.27	1.67	1.39	0.23
**Mo et al. (21)**	CN	155	54	0.13	1.31	0.89	0.23
**Kim et al. (25)**	KR	28	42.6	0.83	0.00	0.78	0.00
**Goyal et al. (30)**	US	393	62.2	0.24	1.08	2.49	0.80
**CDC**	US	7162		0.07	0.03		
**Gold et al. (36)**	US	305	60	0.26	1.47	4.01	0.82
**Miyara et al. (1)**[1]	FR	341		0.24	1.29	3.64	1.05
**Miyara et al. (1)**[2]	FR	132		0.39	0.37	0.66	0.19
**Miyara et al. (1)**[3]	FR	473		0.28	1.04	2.78	0.80
**Han et al. (31)**	CN	17	40	1.00	0.33	1.08	0.49
**Jin et al. (32)**	CN	651	~45	0.23	0.66	0.68	0.01
**Lian et al. (33).**	CN	788		0.24	0.69	0.66	0.03
**Yao et al. (34)**	CN	108	52	0.17	0.83	0.43	0.45**
**Zhang, Ouyang et al. (35)**	CN	120	45.4	0.25	0.90	0.54	0.28

*Mean or median;

** comprises bronchiectasis, COPD, and asthma

[1] hospitalized patients; [2] non-hospitalized patients; [3] all patients;

PR: prevalence ratio; obs.: observed; y: years; HT: hypertension

References

1. Miyara M, Tubach F, Pourcher V, Morelot-Panzini C, Pernet J, Haroche J. Low incidence of daily active tobacco smoking in patients with symptomatic COVID-19. Qeios [Internet]. 2020; published online: 21.04.2020; Date Accessed: 05.05.2020; Pages: 13; DOI: https://doi.org/10.32388/WPP19W.3. Available from: https://www.qeios.com/read/WPP19W.3.

2. Arrêté du 23 avril 2020 complétant l'arrêté du 23 mars 2020 prescrivant les mesures d'organisation et de fonctionnement du système de santé nécessaires pour faire face à l'épidémie de covid-19 dans le cadre de l'état d'urgence sanitaire, NOR : SSAZ2010368A (2020).

3. Protard M. France restricts sales of nicotine substitutes after coronavirus study; 2020. Available from: https://www.reuters.com/article/us-health-coronavirus-france-nicotine/france-restricts-sales-of-nicotine-substitutes-after-coronavirus-study-idUSKCN2262K8.

4. Wan Y, Shang J, Graham R, Baric RS, Li F. Receptor Recognition by the Novel Coronavirus from Wuhan: an Analysis Based on Decade-Long Structural Studies of SARS Coronavirus. J Virol. 2020;94(7):e00127-00120.

5. Alifano M, Alifano P, Forgez P, Iannelli A. Renin-angiotensin system at the heart of COVID-19 pandemic. Biochimie. 2020;174:30-33.

6. Rivellese F, Prediletto E. ACE2 at the centre of COVID-19 from paucisymptomatic infections to severe pneumonia. Autoimmun Rev. 2020;19(6):102536.

7. Shang J, Ye G, Shi K, Wan Y, Luo C, Aihara H, et al. Structural basis of receptor recognition by SARS-CoV-2. Nature. 2020;581(7807):221-224.

8. Nehme A, Zouein FA, Zayeri ZD, Zibara K. An Update on the Tissue Renin Angiotensin System and Its Role in Physiology and Pathology. J Cardiovasc Dev Dis. 2019;6(2):14.

9. Oakes JM, Fuchs RM, Gardner JD, Lazartigues E, Yue X. Nicotine and the renin-angiotensin system. American Journal of Physiology - Regulatory Integrative and Comparative Physiology. 2018;315(5):R895-R906.

10. Aztatzi-Aguilar OG, Uribe-Ramírez M, Arias-Montaño JA, Barbier O, De Vizcaya-Ruiz A. Acute and subchronic exposure to air particulate matter induces expression of angiotensin and bradykinin-related genes in the lungs and heart: Angiotensin-II type-I receptor as a molecular target of particulate matter exposure. Part Fibre Toxicol. 2015;12:17-17.

11. Leung JM, Yang CX, Tam A, Shaipanich T, Hackett T-L, Singhera GK, et al. ACE-2 Expression in the Small Airway Epithelia of Smokers and COPD Patients: Implications for COVID-19. Eur Respir J [Internet]. 2020:2000688; DOI: 10.1183/13993003.00688-2020; Available from: https://erj.ersjournals.com/content/erj/early/2020/03/26/13993003.00688-2020.full.pdf.

12. Brake SJ, Barnsley K, Lu W, McAlinden KD, Eapen MS, Sohal SS. Smoking Upregulates Angiotensin-Converting Enzyme-2 Receptor: A Potential Adhesion Site for Novel Coronavirus SARS-CoV-2 (Covid-19). Journal of Clinical Medicine. 2020;9(3):841.

13. Guan WJ, Liang WH, Zhao Y, Liang HR, Chen ZS, Li YM, et al. Comorbidity and its impact on 1590 patients with Covid-19 in China: A Nationwide Analysis. European Respiratory Journal [Internet]. 2020; published online: 26.03.2020; Date Accessed: 05.05.2020; Pages: 56; DOI: 10.1183/13993003.00547-2020. Available from: https://erj.ersjournals.com/content/early/2020/03/17/13993003.00547-2020.

14. Guan WJ, Ni ZY, Hu Y, Liang WH, Ou CQ, He JX, et al. Clinical Characteristics of Coronavirus Disease 2019 in China. N Engl J Med. 2020;382:1708-1720.

15. Yang X, Yu Y, Xu J, Shu H, Xia Ja, Liu H, et al. Clinical course and outcomes of critically ill patients with SARS-CoV-2 pneumonia in Wuhan, China: a single-centered, retrospective, observational study. The Lancet Respiratory Medicine. 2020;8(5):475-481.

16. Zhang J-j, Dong X, Cao Y-y, Yuan Y-d, Yang Y-b, Yan Y-q, et al. Clinical characteristics of 140 patients infected with SARS-CoV-2 in Wuhan, China. Allergy.75:1730-1741.

17. Liu W, Tao ZW, Wang L, Yuan ML, Liu K, Zhou L, et al. Analysis of factors associated with disease outcomes in hospitalized patients with 2019 novel coronavirus disease. Chin Med J (Engl). 2020;133(9):1032-1038.

18. Huang C, Wang Y, Li X, Ren L, Zhao J, Hu Y, et al. Clinical features of patients infected with 2019 novel coronavirus in Wuhan, China. The Lancet. 2020;395(10223):497-506.

19. Chen T, Wu D, Chen H, Yan W, Yang D, Chen G, et al. Clinical characteristics of 113 deceased patients with coronavirus disease 2019: retrospective study. BMJ. 2020;368:m1091.

20. Zhou F, Yu T, Du R, Fan G, Liu Y, Liu Z, et al. Clinical course and risk factors for mortality of adult inpatients with COVID-19 in Wuhan, China: a retrospective cohort study. The Lancet. 2020;395(10229):1054-1062.

21. Mo P, Xing Y, Xiao Y, Deng L, Zhao Q, Wang H, et al. Clinical characteristics of refractory COVID-19 pneumonia in Wuhan, China. Clin Infect Dis [Internet]. 2020:ciaa270; DOI: 10.1093/cid/ciaa270; Available from: https://academic.oup.com/cid/advance-article/doi/10.1093/cid/ciaa270/5805508.
22. Wan S, Xiang Y, Fang W, Zheng Y, Li B, Hu Y, et al. Clinical features and treatment of COVID-19 patients in northeast Chongqing. J Med Virol. 2020;92:797-806.
23. Zhang X, Cai H, Hu J, Lian J, Gu J, Zhang S, et al. Epidemiological, clinical characteristics of cases of SARS-CoV-2 infection with abnormal imaging findings. International Journal of Infectious Diseases. 2020;94:81-87.
24. Shi Y, Yu X, Zhao H, Wang H, Zhao R, Sheng J. Host susceptibility to severe COVID-19 and establishment of a host risk score: findings of 487 cases outside Wuhan. Critical Care. 2020;24(1):108.
25. Kim ES, Chin BS, Kang CK, Kim NJ, Kang YM, Choi J-P, et al. Clinical Course and Outcomes of Patients with Severe Acute Respiratory Syndrome Coronavirus 2 Infection: a Preliminary Report of the First 28 Patients from the Korean Cohort Study on COVID-19. J Korean Med Sci. 2020;35(13):e142.
26. Li X, Xu S, Yu M, Wang K, Tao Y, Zhou Y, et al. Risk factors for severity and mortality in adult COVID-19 inpatients in Wuhan. J Allergy Clin Immunol. 2020;146(1):110-118.
27. Wang R, Pan M, Zhang X, Han M, Fan X, Zhao F, et al. Epidemiological and clinical features of 125 Hospitalized Patients with COVID-19 in Fuyang, Anhui, China. International Journal of Infectious Diseases. 2020;95:421-428.
28. Feng Y, Ling Y, Bai T, Xie Y, Huang J, Li J, et al. COVID-19 with Different Severity: A Multi-center Study of Clinical Features. Am J Respir Crit Care Med. 2020;201(11):1380-1388.
29. Ji D, Zhang D, Xu J, Chen Z, Yang T, Zhao P, et al. Prediction for Progression Risk in Patients with COVID-19 Pneumonia: the CALL Score. Clin Infect Dis [Internet]. 2020:ciaa414; DOI: 10.1093/cid/ciaa414; Available from: https://academic.oup.com/cid/advance-article/doi/10.1093/cid/ciaa414/5818317.
30. Goyal P, Choi JJ, Pinheiro LC, Schenck EJ, Chen R, Jabri A, et al. Clinical Characteristics of Covid-19 in New York City. New Engl J Med. 2020;382:2372-2374.
31. Han X, Cao Y, Jiang N, Chen Y, Alwalid O, Zhang X, et al. Novel Coronavirus Pneumonia (COVID-19) Progression Course in 17 Discharged Patients: Comparison of Clinical and Thin-Section CT Features During Recovery. Clin Infect Dis. 2020;71(15):723-731.
32. Jin X, Lian J-S, Hu J-H, Gao J, Zheng L, Zhang Y-M, et al. Epidemiological, clinical and virological characteristics of 74 cases of coronavirus-infected disease 2019 (COVID-19) with gastrointestinal symptoms. Gut. 2020;69(6):1002-1009.
33. Lian J, Jin X, Hao S, Cai H, Zhang S, Zheng L, et al. Analysis of Epidemiological and Clinical features in older patients with Corona Virus Disease 2019 (COVID-19) out of Wuhan. Clin Infect Dis. 2020;71(15):740-747.
34. Yao Q, Wang P, Wang X, Qie G, Meng M, Tong X, et al. Retrospective study of risk factors for severe SARS-Cov-2 infections in hospitalized adult patients. Pol Arch Intern Med. 2020;130:390-399.
35. Zhang R, Ouyang H, Fu L, Wang S, Han J, Huang K, et al. CT features of SARS-CoV-2 pneumonia according to clinical presentation: a retrospective analysis of 120 consecutive patients from Wuhan city. Eur Radiol. 2020;30:4417-4426.
36. Gold JAW, Wong KK, Szablewski CM, Patel PR, John R, da Silva J, et al. Characteristics and Clinical Outcomes of Adult Patients Hospitalized with COVID-19 — Georgia, March 2020. Morb Mortal Weekly Rep. 2020;69.
37. CDC COVID-19 Response Team. Preliminary Estimates of the Prevalence of Selected Underlying Health Conditions Among Patients with Coronavirus Disease 2019 — United States, February 12–March 28, 2020. Morb Mortal Weekly Rep [Internet]. 2020;69(13):[382-386 pp.]; Available from: https://www.cdc.gov/mmwr/volumes/69/wr/pdfs/mm6913e2-H.pdf.
38. Vardavas CI, Nikitara K. COVID-19 and smoking: A systematic review of the evidence. Tobacco induced diseases. 2020;18:20.
39. Lippi G, Henry BM. Active smoking is not associated with severity of coronavirus disease 2019 (COVID-19). Eur J Intern Med. 2020;75:107-108.
40. Zhao Q, Meng M, Kumar R, Wu Y, Huang J, Lian N, et al. The impact of COPD and smoking history on the severity of Covid-19: A systemic review and meta-analysis. J Med Virol. 2020:1-7.
41. Gonzalez-Rubio J, Navarro-Lopez C, Lopez-Najera E, Lopez-Najera A, Jiménez-Díaz L, Navarro-López J, et al. What is Happening with Smokers and COVID-19? A Systematic Review and a Meta-Analysis. Preprints.org [Internet]. 2020; published online: 30.04.2020; Date Accessed: 31.08.2020; Pages: 12; DOI: 10.20944/preprints202004.0540.v1. Available from: https://www.preprints.org/manuscript/202004.0540/v1.

42. Farsalinos K, Barbouni A, Niaura R. Systematic review of the prevalence of current smoking among hospitalized COVID-19 patients in China: could nicotine be a therapeutic option? Internal and Emergency Medicine. 2020;15:845-852.
43. Farsalinos K, Poulas K, Polosa R, Barbouni A, Caponnetto P, Niaura R. Prevalence of Current Smoking and Association with Adverse Outcome in Hospitalized COVID-19 Patients: A Systematic Review and Meta-Analysis. Preprints.org [Internet]. 2020; published online: 07.05.2020; Date Accessed: 31.08.2020; DOI: doi: 10.20944/preprints202005.0113.v1. Available from: https://www.preprints.org/manuscript/202005.0113/v1.
44. Simons D, Shahab L, Brown J, Perski O. The association of smoking status with SARS-CoV-2 infection, hospitalisation and mortality from COVID-19: A living rapid evidence review (version 6). Qeios [Internet]. 2020; published online: 02.08.2020; Date Accessed: 31.08.2020; Pages: 47; DOI: 10.32388/UJR2AW.7. Available from: https://www.qeios.com/read/UJR2AW.7.
45. Wang Z, Chen Z, Zhang L, Wang X, Hao G, Zhang Z, et al. Status of Hypertension in China. Circulation. 2018;137(22):2344-2356.
46. Gorban de Lapertosa S, Ferreira de Moura A, Decroux C, Duke L, Hammond L, Jacobs E, et al. IDF Diabetes Atlas 9th Edition: International Diabetes Federation; 2019. Available from: https://www.diabetesatlas.org/en/resources/.
47. Virani SS, Alonso A, Benjamin EJ, Bittencourt MS, Callaway CW, Carson AP, et al. Heart Disease and Stroke Statistics - 2020 Update: A Report From the American Heart Association. Circulation. 2020;141(9):e139-e596.
48. Fang L, Gao P, Bao H, Tang X, Wang B, Feng Y, et al. Chronic obstructive pulmonary disease in China: a nationwide prevalence study. The Lancet Respiratory Medicine. 2018;6(6):421-430.
49. Hwang YI, Park YB, Yoo KH. Recent Trends in the Prevalence of Chronic Obstructive Pulmonary Disease in Korea. Tuberc Respir Dis (Seoul). 2017;80(3):226-229.
50. Centers for Disease Control and Prevention. Chronic Obstructive Pulmonary Disease (COPD) Fact Sheets; Date Accessed: 11.06.2020. Available from: https://www.cdc.gov/copd/maps/index.html.
51. Roche N, Zureik M, Vergnenègre A, Huchon G, Neukirch F. Données récentes sur la prévalence de la bronchopneumopathie chronique obstructive en France. Bulletin épidemiologique hebdomadaire. 2007(27-28):245-248.
52. Biener AI, Decker SL, Rohde F. Prevalence and Treatment of Chronic Obstructive Pulmonary Disease (COPD) in the United States. JAMA. 2019;322(7):602-602.
53. Creamer MR, Wang TW, Babb S, Cullen KA, Day H, Willis G, et al. Tobacco Product Use and Cessation Indicators Among Adults — United States, 2018. Morb Mortal Weekly Rep. 2019;68(45):1013-1019.
54. Andler R, Richard J-B, Guignard R, Quatremère G, Verrier F, Gane J, et al. Baisse de la prévalence du tabagisme quotidien parmi les adultes: Résultats du Baromètre de Santé publique France 2018 Bulletin épidémiologique hebdomadaire. 2019(15):271-277.
55. Parascandola M, Xiao L. Tobacco and the lung cancer epidemic in China. Transl Lung Cancer Res. 2019;8(Suppl 1):S21-S30.
56. Won S. Share of cigarette smokers in South Korea from 2008 to 2018, by gender Statista.com; 2020; updated: 31.03.2020; Date Accessed: 11.06.2020. Available from: https://www.statista.com/statistics/645282/south-korea-smoking-rate-by-gender/.
57. Perrine A-L, Lecoffre C, Blacher J, Olié V. L'hypertension artérielle en Franve: prévalence, traitement et controle en 2015 et évolutions depuis 2006. Bulletin épidémiologique hebdomadaire. 2018(10):170-179.
58. The Korean Society Hypertension (KSH) – Hypertension Epidemiology Research Working Group, Kim HC, Cho MC. Korea hypertension fact sheet 2018. Clin Hypertens. 2018;24:13.

List of tables

Table 1: Overview on meta-analyses 6

Table 2: Observed and expected smoker, prevalence ratio (PR), and probability that observed population is representative for general population 8

Table 3: Smoking prevalence and prevalence of comorbidities in the countries of the studied cohorts 10

Table 4: Compilation of prevalence ratios (PR) of the observed prevalence with the prevalence in the respective general population 12

GETTING IN TOUCH WITH THE EU

In person

All over the European Union there are hundreds of Europe Direct information centres. You can find the address of the centre nearest you at: https://europa.eu/european-union/contact_en

On the phone or by email

Europe Direct is a service that answers your questions about the European Union. You can contact this service:

- by freephone: 00 800 6 7 8 9 10 11 (certain operators may charge for these calls),

- at the following standard number: +32 22999696, or

- by electronic mail via: https://europa.eu/european-union/contact_en

FINDING INFORMATION ABOUT THE EU

Online

Information about the European Union in all the official languages of the EU is available on the Europa website at: https://europa.eu/european-union/index_en

EU publications

You can download or order free and priced EU publications from EU Bookshop at: https://publications.europa.eu/en/publications. Multiple copies of free publications may be obtained by contacting Europe Direct or your local information centre (see https://europa.eu/european-union/contact_en).

KJ-NA-30373-EN-N

The European Commission's science and knowledge service

Joint Research Centre

JRC Mission

As the science and knowledge service of the European Commission, the Joint Research Centre's mission is to support EU policies with independent evidence throughout the whole policy cycle.

EU Science Hub
ec.europa.eu/jrc

@EU_ScienceHub

EU Science Hub - Joint Research Centre

EU Science, Research and Innovation

EU Science Hub

Publications Office
of the European Union

doi:10.2760/564217

ISBN 978-92-76-22062-6
